# Pan-cancer analysis on the role of PIK3R1 and PIK3R2 in human tumors

**DOI:** 10.1038/s41598-022-09889-0

**Published:** 2022-04-08

**Authors:** Yane Liu, Duo Wang, Zhijun Li, Xinwei Li, Mengdi Jin, Ningning Jia, Xingyao Cui, Guoyan Hu, Tongyu Tang, Qiong Yu

**Affiliations:** 1 Department of Epidemiology and Biostatistics, School of Public Health, Jilin University, Changchun, 130021 Jilin China; 2 Department of Hand and Foot Surgery, The First Hospital of Jilin University, Changchun, 130021 Jilin China; 3 Department of Gastroenterology, The First Hospital of Jilin University, Changchun, 130021 Jilin China

**Keywords:** Cancer, Computational biology and bioinformatics

## Abstract

Phosphoinositide-3-Kinase Regulatory Subunit 1 (PIK3R1) is believed to function as a tumor suppressor, while Phosphoinositide-3-Kinase Regulatory Subunit 2 (PIK3R2) as a tumor driver. However, there is no systematic pan-cancer analysis of them. The pan-cancer study comprehensively investigated the gene expression, genetic alteration, DNA methylation, and prognostic significance of PIK3R1 and PIK3R2 in 33 different tumors based on the TIMER, GEPIA, UALCAN, HPA, cBioPortal, and Kaplan–Meier Plotter database. The results indicated that PIK3R1 is lowly expressed in most tumors while PIK3R2 is highly expressed in most tumors, and abnormal gene expression may be related to promoter methylation. Moreover, not only mutations, downregulation of PIK3R1 and upregulation of PIK3R2 were found to be detrimental to the survival of most cancer patients as well. Furthermore, the expression of both PIK3R1 and PIK3R2 was associated with the level of immune infiltration in multiple tumors, such as breast invasive carcinoma. Our study conducted a comparatively comprehensive analysis of the role of PIK3R1 and PIK3R2 in a variety of cancers, contributing to further study of their potential mechanisms in cancer occurrence and progression. Our findings suggested that PIK3R1 and PIK3R2 could serve as prognostic markers for several cancers.

## Introduction

Cancer is one of the most serious diseases that threatens human health, and the disease burden of tumors is on the rise both in worldwide countries and ours^1^. Therefore, efforts to elucidate molecular mechanisms of tumorigenesis and explore potential biomarkers for cancer diagnosis and prediction of cancer treatment outcomes are particularly important for cancer prevention and control. Based on developing techniques and multiple public resources, bioinformatics approaches allow us to perform pan-cancer analysis for investigating cancer-related genes of interest.

Phosphoinositide 3-kinase (PI3K) activity is stimulated by diverse oncogenes and growth factor receptors, and aberrations in PI3K signaling are considered a hallmark of cancer^2^. PI3K enzymes, a conserved family of lipid kinases composed of a catalytic subunit and a regulatory subunit, are divided into three categories according to different structures, of which class I PI3K is most characterized^3^. PIK3R1 (Phosphoinositide-3-Kinase Regulatory Subunit 1), which encodes the predominant regulatory subunit P85α of class I PI3K, inhibits the catalytic activity of P110α kinase and combines directly with PTEN and enhances lipid phosphatase activity. On the other hand, PIK3R2 (Phosphoinositide-3-Kinase Regulatory Subunit 2) encodes the ubiquitous regulatory p85β of class I PI3K, interacts with tyrosine kinase (signal transduction), and is an important molecule of PI3K pathway. An increasing number of studies have revealed that PIK3R1 and PIK3R2 are aberrantly expressed in tumors and are associated with increased cell proliferation and invasion as well as reduced apoptosis^4,5^. Moreover, PIK3R1 and PIK3R2 are widely expressed and frequently mutated. It has been confirmed that tumor mutations in PIK3R1 were associated with enhanced PI3K signaling through weakening the ability of p85α to inhibit p110α^6^. Although PIK3R1 and PIK3R2 have been shown to serve opposite roles as tumor-suppressor gene and oncogenes in multiple tumors, evidence of their role in pan-cancer is lacking.

In the present study, the transcriptional expression levels of PIK3R1 and PIK3R2 genes in pan-cancer were first visualized using the TIMER and GEPIA database. Additionally, we analyzed the correlation of gene expression with prognosis and immune infiltration via Kaplan-Meier plotter and TIMER database. We also considered genetic alteration, DNA methylation, and enrichment pathways of PIK3R1 and PIK3R2. This analysis demonstrated potential molecular mechanisms of PIK3R1 and PIK3R2 in tumorigenesis and clinical prognosis of various human tumors. Meanwhile, it suggested that besides gene mutations, decreased or increased mRNA expression levels of PIK3R1 and PIK3R2 are supposed to be considered in clinical management of cancer.

## Materials and methods

### Sample information

The original data of the public databases that used for systematic pan-cancer analysis of PIK3R1 and PIK3R2 were mainly from The Cancer Genome Atlas (TCGA) (https://cancergenome.nih.gov/) and detailed clinical information of 33 TCGA tumors was displayed in Supplementary Table S1. Full name of the tumors and corresponding abbreviations were given below: Adrenocortical carcinoma (ACC); Bladder Urothelial Carcinoma (BLCA); Breast invasive carcinoma (BRCA); Cervical squamous cell carcinoma and endocervical adenocarcinoma (CESC); Cholangiocarcinoma (CHOL); Colon adenocarcinoma (COAD); Lymphoid Neoplasm Diffuse Large B-cell Lymphoma (DLBC); Esophageal carcinoma (ESCA); Glioblastoma multiforme (GBM); Head and Neck squamous cell carcinoma (HNSC); Kidney Chromophobe (KICH); Kidney renal clear cell carcinoma (KIRC); Kidney renal papillary cell carcinoma (KIRP); Acute Myeloid Leukemia (LAML); Brain Lower Grade Glioma (LGG); Liver hepatocellular carcinoma (LIHC); Lung adenocarcinoma (LUAD); Lung squamous cell carcinoma (LUSC); Mesothelioma (MESO); Ovarian serous cystadenocarcinoma (OV); Pancreatic adenocarcinoma (PAAD); Pheochromocytoma and Paraganglioma (PCPG); Prostate adenocarcinoma (PRAD); Rectum adenocarcinoma (READ); Sarcoma (SARC); Skin Cutaneous Melanoma (SKCM); Stomach adenocarcinoma (STAD); Testicular Germ Cell Tumors (TGCT); Thyroid carcinoma (THCA); Thymoma (THYM); Uterine Corpus Endometrial Carcinoma (UCEC); Uterine Carcinosarcoma (UCS); Uveal Melanoma (UVM).

### Transcriptional expression analysis of genes

TIMER 2.0 (http://timer.cistrome.org/) database is an ideal resource for the systematic analysis of associations between gene expression and tumor features in TCGA^7^. The transcriptional expression profiling of PIK3R1 and PIK3R2 were compared between tumor types and the corresponding normal tissues using the Gene_DE Module of TIMER. For some tumors lacking normal tissues, we used the Expression Analysis Module of GEPIA2 (http://gepia2.cancer-pku.cn), whose data comes from TCGA and Genotype-Tissue Expression (GTEx) databases^8^, to obtain box plots of the gene expression between these tumors and normal tissues. It was worth mentioning that GTEx covers more than 7000 samples from 449 healthy human donors, which can effectively make up for the lack of normal tissue of TCGA^9^, and Supplementary Table S2 summarized the tissue types and sample sizes covered by the database. Moreover, GEPIA was also used to explore the expression of PIK3R1 and PIK3R2 in different pathological stages of tumors from TCGA database.

### Proteomic expression analysis of genes

The UALCAN database (http://ualcan.path.uab.edu/analysis-prot.html), provides protein expression analysis of six tumor types, including BRCA, COAD, KIRC, LUAD, OV, and UCEC using data from Clinical Proteomic Tumor Analysis Consortium (CPTAC)^10^. And Supplementary Table S3 summarized the sample information of six tumors. The Human Protein Atlas (HPA) (https://www.proteinatlas.org) database, in which researchers used highly specific antibodies and immunoassay technology to detect the expression of 26,000 kinds of human proteins in 64 cell lines, 48 kinds of human normal tissues and 20 kinds of tumor tissues, allows us to obtain Immunohistochemistry (IHC) images of PIK3R1 and PIK3R2 proteomic expression in tumor and normal samples^11^. Results from the two databases were combined to analyze the genetic expression at the protein level.

### Survival prognosis analysis

The Kaplan-Meier plotter (https://kmplot.com/analysis), which is mainly based on Affymetrix microarray information from TCGA databases, can evaluate the effect of 54 k genes on survival in 21 cancer types^12^. The prognostic value of PIK3R1 and PIK3R2 expression in 21 cancers was evaluated by DFS (disease-free survival), DMFS (distant metastasis-free survival), FP (first progression survival), OS (overall survival), PPS (relapse survival), and RFS (relapse-free survival).

### Genetic alteration analysis

Genetic alteration data of PIK3R1 and PIK3R2, including alteration frequency, mutation type, mutated site, were available from the cBioPortal database, which is an open platform for exploring multidimensional cancer genomics data^13,14^. The Cancer Types Summary Module was used to obtain the alteration frequency, mutation type of genes in TCGA tumors. The detailed information of gene mutations and the impact of gene mutations on the survival of cancer patients were obtained through Mutations Module and Survival Module, respectively.

### DNA methylation analysis

The UALCAN online tool can evaluate epigenetic regulation of gene expression by promoter methylation based on the TCGA database^10^. Consequently, the DNA methylation of PIK3R1 and PIK3R2 were analyzed using UALCAN database.

### Gene-immune analysis

Tumor Mutational Burden (TMB) and Microsatellite Instability (MSI) are considerable biomarkers in tumor microenvironment, so SangerBox (http://sangerbox.com/Tool), a practical and free online tool for analyzing TCGA data, was used to study the relationships between gene expression and TMB, MSI, and Immune Checkpoint (ICP) Genes. A total of 47 ICP genes were analyzed for their relationship with gene expression, and spearman’s rank test was used in the above correlation analysis.

### Immune infiltration analysis

The Gene module of the TIMER database and SangerBox were used to visualize the correlation of gene expression with immune infiltration levels in tumors of TCGA, and types of the immune cells included B cells, CD4 + T cells, CD8 + T cells, neutrophils, macrophages, dendritic cells and cancer-associated fibroblast.

### Protein–protein interaction (PPI) networks and enrichment analysis

The protein–protein interaction (PPI) networks of PIK3R1 and PIK3R2 were acquired through the STRING database^15^. We input PIK3R1 and PIK3R2 in the Multiple proteins, and basic settings were as follows: active interaction sources (experiment), the minimum required interaction score (low confidence (0.150)), and max number of interactors to show (no more than 50 interactors). Thereafter, we utilized genes that interact with PIK3R1 and PIK3R2 which were obtained from the STRING database, to perform enrichment analysis. Functional enrichment of interacting genes, including BP (biological process), CC (cellular component), and MF (molecular function), and KEGG pathway (www.kegg.jp/kegg/kegg1.html)^16^, were gained from the DAVID database (https://david.ncifcrf.gov). And then files of the functional annotation chart were downloaded. Eventually, the obtained results were visualized with R 4.0.5 and Cytoscape software.

### Statistical analysis

The Wilcoxon test was used to evaluated the differential expression between tumors and adjacent normal tissues in TIMER. The Mann–Whitney U test was used to analyze the IHC results that obtained in HPA database. To compare survival curves, the log rank test to calculate the HR and logrank P-value in Kaplan–Meier Plotter. The correlation of gene expression was analyzed using Spearman’s correlation. What’s more, *P* < 0.05 was considered as statistically significant, and statistical analyses were performed using the statistical software SPSS 24.0. The alpha level for all tests was 0.05.

### Ethical approval

All experimental protocols were conducted in accordance with relevant guidelines and regulations.

## Results

### Expression levels of PIK3R1 and PIK3R2 in pan-cancer

**Figure 1:**
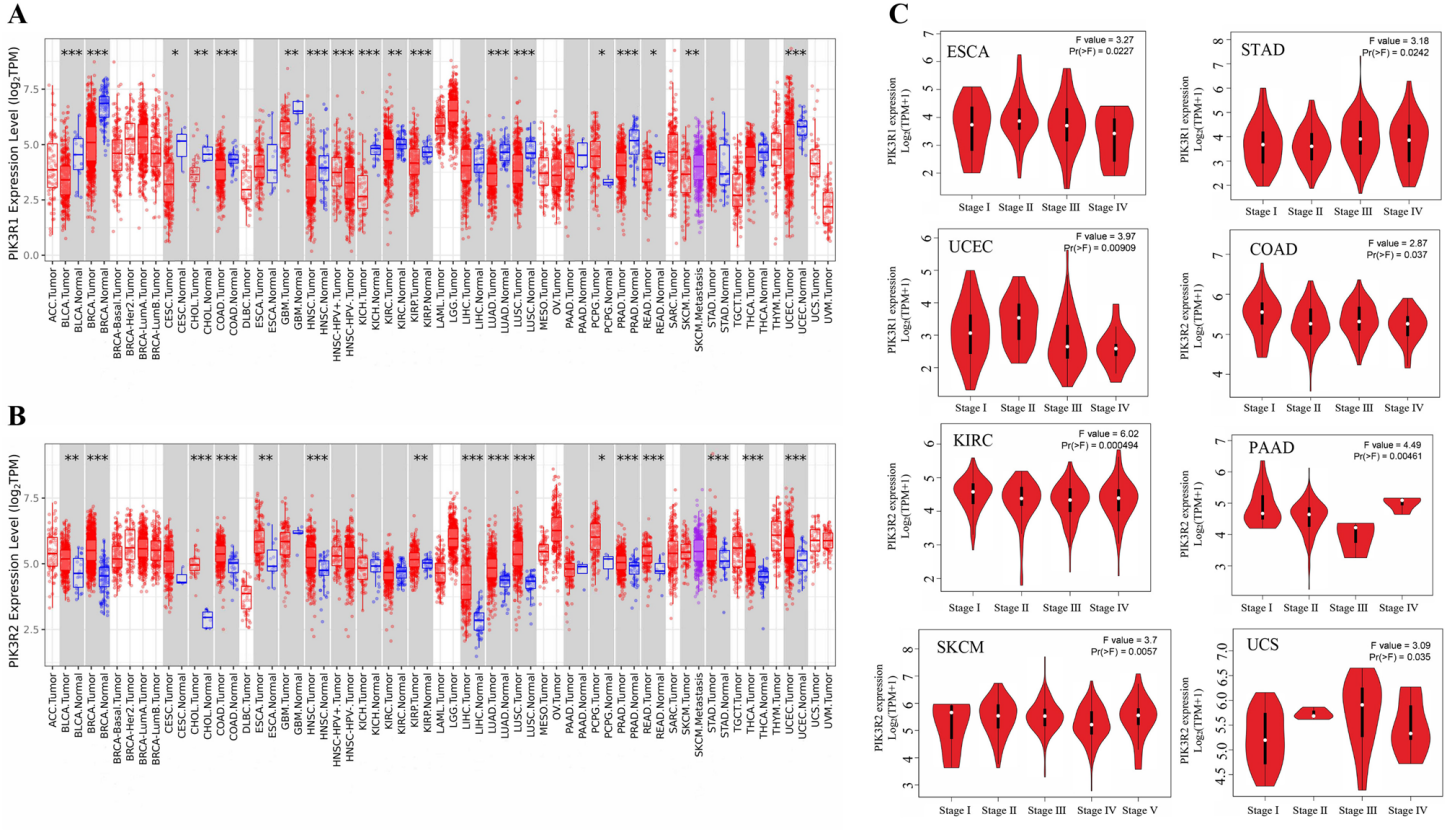
Expression levels of PIK3R1 and PIK3R2 in different tumors and pathological stages. (**A**) Expression levels of PIK3R1 in different tumors vs corresponding controls were analyzed by TIMER 2.0 (http://timer.cistrome.org/database). * P < 0.05; ** P < 0.01; *** P < 0.001. (**B**) Expression levels of PIK3R2 in different tumors vs corresponding controls were analyzed by TIMER 2.0. * P < 0.05; ** P < 0.01; *** P < 0.001. (**C**) PIK3R1 expression levels were compared among main pathological stages (stage I, stage II, stage III, stage IV) of ESCA, STAD, UCEC, and PIK3R2 expression levels were compared between different stages of COAD, KIRC, PAAD, SKCM, UCS. Genetic expression level was shown as log2 (TPM + 1). Analyses were conducted via the GEPIA 2 database (http://gepia2.cancer-pku.cn).

We first used the TIMER database to analyze the expression levels of PIK3R1 and PIK3R2. As shown in Fig. 1A, the expression level of PIK3R1 was significantly lower in tumors than in the corresponding normal tissues including BLCA, BRCA, COAD, KICH, KIRC, KIRP, HNSC, LUAD, LUSC, PRAD, UCEC (*P* < 0.001), CHOL, GBM, SKCM (*P* < 0.01), READ, CESC (*P* < 0.05). In contrast, PIK3R1 showed higher expression in HNSC-HPV positive (*P* < 0.001), PCPG (*P* < 0.05) than in their corresponding control tissues. Furthermore, the expression level of PIK3R2 was significantly higher in BRCA, CHOL, HNSC, LIHC, LUAD, LUSC, PRAD, READ, STAD, THCA, UCEC (*P* < 0.001), BLCA, ESCA, KIRC (*P* < 0.01), PCPG (*P* < 0.05) than in the corresponding control tissues (Fig. 1B).

We further used the GEPIA database to evaluate the expression differences of PIK3R1 and PIK3R2 between the tumor and the control tissues as the corresponding control data was not available in the TIMER database for some tumors. As shown in Fig. S1A, PIK3R1 showed a lower expression in OV and UCS (*P* < 0.05) than in normal tissue but a higher expression in LAML and THYM (*P* < 0.05) than in normal tissues. However, we didn’t observe significant differences for other tumors, such as ACC, DLBC, LGG, SARC, or TGCT. As for PIK3R2, we found it highly expressed in TGCT, UCS, and DLBC (*P* < 0.05), while no significant differences were obtained in ACC, LAML, LGG, OV, SARC (Fig. S1B). Overall, PIK3R1 expression was lower in most cancers while PIK3R2 was higher compared with the corresponding control tissue.

Moreover, according to the “stage plot” module of GEPIA, we observed that PIK3R1 expression in ESCA, STAD, and UCEC (*P* < 0.05) was related to tumor stage while no association was found in other cancers. Meanwhile, PIK3R2 expression in COAD, KIRC, PAAD, SKCM, UCS (*P* < 0.05) was associated with tumor stages, but not others (Fig. 1C).

In addition to analyzing genetic expression at the transcriptional level, we also used the UALCAN database to analyze the proteomic expression levels of genes in different tumors. We found that PIK3R1 proteomic expression in BRCA, COAD, LUAD, OV, and UCEC was significantly lower compared to normal samples, but higher in KIRC (Fig. S2A, *P* < 0.05). Concurrently, PIK3R2 proteomic expression was significantly higher in KIRC and UCEC, but lower in COAD and LUAD (Fig. S2B, *P* < 0.05). Besides, we used the HPA database to explore the protein expression levels of PIK3R1 and PIK3R2 genes through immunohistochemistry images. The results revealed that PIK3R1 and PIK3R2 showed medium staining in most normal tissues, while in most tumor tissues PIK3R1 showed low or negative staining and PIK3R2 showed medium or high staining. Notably, the protein levels of PIK3R1 in cervical cancer, renal cancer, lung cancer, skin cancer, stomach cancer, and testis cancer were lower compared to normal tissues, which is consistent with the results from transcriptional analysis (Fig. S3A). Meanwhile, we also observed a higher proteomic expression level of PIK3R2 in liver cancer and ovarian cancer compared to normal tissues (Fig. S3B). The statistical analysis results of differences in the expression of PIK3R1 and PIK3R2 in normal and tumor tissues were shown in Fig. S3C,D. Although statistical analysis displayed that the expression differences of PIK3R1 and PIK3R2 in lung cancer, stomach cancer, or other cancers were not statistically significant, which may be due to the small sample size, their abnormal expression in cancer was distinct.

### Prognostic value of PIK3R1 and PIK3R2 in different cancers

**Figure 2:**
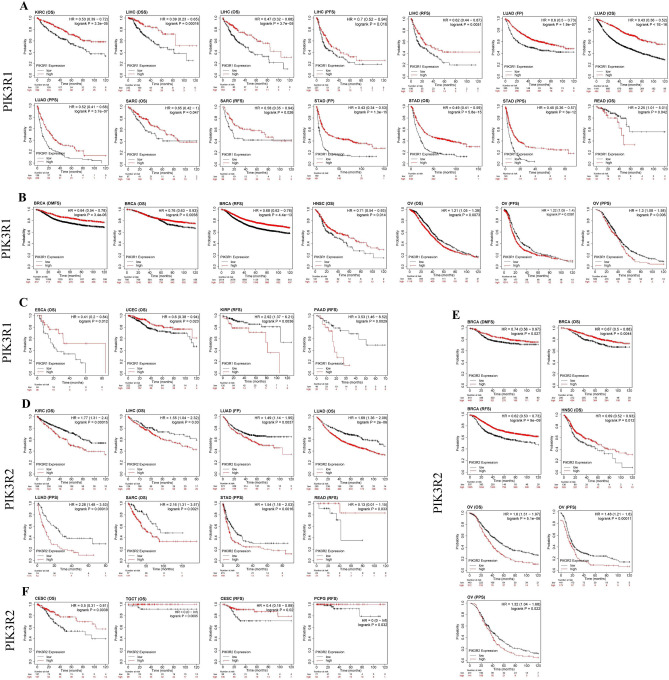
Survival curves comparing low and high expressions of PIK3R1 and PIK3R2 in different tumors. (**A**) Correlations between PIK3R1 expression levels and patient survival in KIRC, LIHC, LUAD, SARC, STAD, READ were analyzed using Kaplan–Meier Plotter database (https://kmplot.com/analysis). (**B**) Correlations between PIK3R1 expression levels and patient survival in BRCA, HNSC, OV. (**C**) Correlations between PIK3R1 expression levels and patient survival in ESCA, UCEC, KIRP, PAAD. (**D**) Correlations between PIK3R2 expression levels and patient survival in KIRC, LIHC, LUAD, SARC, STAD, READ. (**E**) Correlations between PIK3R2 expression levels and patient survival in BRCA, HNSC, OV. (**F**) Correlations between PIK3R2 expression levels and patient survival in CESC, TGCT, PCPG. All the survival analyses were conducted via Kaplan–Meier Plotter database (https://kmplot.com/analysis).

Kaplan–Meier Plotter tool were used to explore the correlation between PIK3R1 and PIK3R2 expression levels with the prognosis of patients with 21 different tumors. Notably, the expression of PIK3R1 and PIK3R2 were significantly correlated with the prognosis of 13 and 12 cancer types, respectively. Furthermore, expression of both PIK3R1 and PIK3R2 was associated with the prognosis of 9 cancer types, including KIRC, LIHC, LUAD, SARC, STAD, READ, OV, BRCA, and HNSC. Among them, PIK3R1 and PIK3R2 plays opposite roles in 6 cancer types and similar roles in 3 cancer types. Specifically, as shown in Fig. 2A,D, lower expressed PIK3R1 was linked to poor prognosis for cancers of KIRC (OS: HR = 0.53, *P* = 3.3e−05), LIHC (DSS: HR = 0.39, *P* = 0.00016; OS: HR = 0.47, *P* = 3.7e−05; PFS: HR = 0.70, *P* = 0.016; RFS: HR = 0.62, *P* = 0.0051), LUAD (FP: HR = 0.6, *P* = 1.9e−07; OS: HR = 0.43, *P* < 1e−16; PPS: HR = 0.52, *P* = 3.7e−07), SARC (OS: HR = 0.65, *P* = 0.047; RFS: HR = 0.58, *P* = 0.026), STAD (FP: HR = 0.43, *P* = 1.3e−15; OS: HR = 0.49, *P* = 5.8e−15; PPS: HR = 0.45, *P* = 3e−12), while higher expression of PIK3R2 predicted poor prognosis for cancers of KIRC (OS: HR = 1.77, *P* = 0.00015), LIHC (OS: HR = 1.55, *P* = 0.03), LUAD (FP: HR = 1.49, *P* = 0.0037; OS: HR = 1.69, *P* = 2e−06; PPS: HR = 2.28, *P* = 0.00013), SARC (OS: HR = 2.16, *P* = 0.0021), STAD (PPS: HR = 1.54, *P* = 0.0016). Interestingly, we also found that lower expression of PIK3R1 and highly expression of PIK3R2 were associated with better prognosis of READ [PIK3R1 (OS: HR = 2.25, *P* = 0.042); PIK3R2 (RFS: HR = 0.13, *P* = 0.033)]. Moreover, as shown in Fig. 2B,E, highly expression of PIK3R1 and PIK3R2 both had protective effect on prognosis for BRCA [PIK3R1 (DMFS: HR = 0.64, *P* = 3.4e−06; OS: HR = 0.76, *P* = 0.0058; RFS: HR = 0.68, *P* = 4.4e−13); PIK3R2 (DMFS: HR = 0.74, *P* = 0.027; OS: HR = 0.67, *P* = 0.0044; RFS: HR = 0.62, *P* = 9e−09)] and HNSC [PIK3R1 (OS: HR = 0.71, *P* = 0.014); PIK3R2 (OS: HR = 0.069, *P* = 0.012)]. However, highly expression of PIK3R1 and PIK3R2 both play detrimental roles in OV [PIK3R1 (OS: HR = 1.21, *P* = 0.0073; PFS: HR = 1.22, *P* = 0.0081; PPS: HR = 1.30, *P* = 0.006); PIK3R2 (OS: HR = 1.6, *P* = 5.1e−06; PFS: HR = 1.48, *P* = 0.00011; PPS: HR = 1.32, *P* = 0.022)]. Specially, we identified highly expression of PIK3R1 as favorable prognostic factors in ESCA (OS: HR = 0.41, *P* = 0.012) and UCEC (OS: HR = 0.6, *P* = 0.023) and as detrimental factors in KIRP (RFS: HR = 2.92, *P* = 0.0036) and PAAD (RFS: HR = 3.53, *P* = 0.0029) (Fig. 2C), while we failed to obtain a correlation between PIK3R2 expression and prognosis in these cancers. Meanwhile, increased PIK3R2 expression predicted better OS for CESC (HR = 0.50, *P* = 0.0038), TGCT (HR = 0, *P* = 0.0095) and better RFS for CESC (HR = 0.4, *P* = 0.02) and PCPG (HR = 0, *P* = 0.032) (Fig. 2F), but we did not observe that PIK3R1 significantly influenced the prognosis of these three cancers. Finally, we failed to obtain correlations between PIK3R1 or PIK3R2 expression and prognosis of other cancers.

### Results of gene alteration analysis

**Figure 3:**
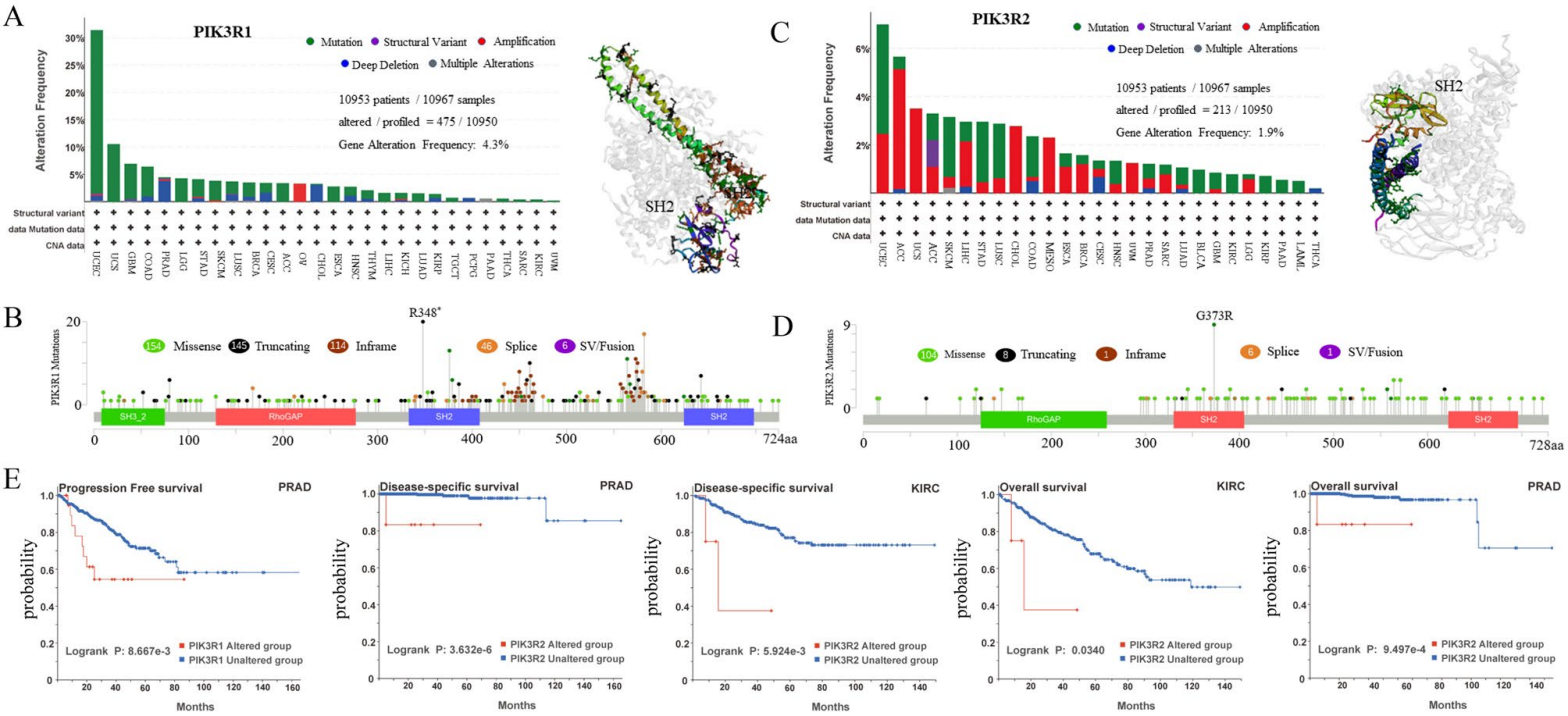
Mutation characteristics of PIK3R1 and PIK3R2 in different tumors of TCGA. The mutation characteristics of genes were explored via the cBioPortal database (https://www.cbioportal.org/). (**A**) The alteration frequency with mutation types in all TCGA tumors and the 3D structure of PIK3R1. (**B**) Sites of different mutation types of PIK3R1. (**C**) The alteration frequency with mutation types in all TCGA tumors and the 3D structure of PIK3R2. (**D**) The sites of different mutation types of PIK3R2. (**E**) Relationship between the mutation status of genes and patient survival.

We further explored the PIK3R1 and PIK3R2 genetic alteration status in human cancers of TCGA cohorts using cBioPortal. As shown in Fig. 3A, of all 10,950 patients, 475 (4%) had PIK3R1 mutations. And in most cancers, “mutation” is the primary alteration type of PIK3R1, and the highest alteration frequency of PIK3R1 (31.38%) occurs in cases with UCEC (Fig. 3A). In addition, 465 PIK3R1 mutations in tumors were distributed throughout the whole sequence of PIK3R1 with 14 statistically significant hotspots mutations and we also found missense and truncating were the two important mutation types of PIK3R1, and R348* alteration, which was the most frequent mutation in the SH2 domain, located in the nucleus where they promote nuclear JNK pathway activation (Fig. 3B). By same methods, as shown in Fig. 3C, of all 10,950 patients, 213 (1.9%) had PIK3R2 mutations. In addition, we observed the highest frequency of PIK3R2 was approximately 7% in the cases with UCEC, and mutation and amplification were the common types among the different types of genetic alterations (Fig. 3C). Moreover, missense mutation was the main type of PIK3R2 genetic mutations and G37R missense mutation was the most frequent mutation (Fig. 3D). As shown in Fig. 3E, we also noticed that PRAD patients with PIK3R1 alterations have poorer prognosis in progression-free survival than those without PIK3R1 alterations (*P* < 0.05). In addition, patients with PIK3R2 alterations showed poorer prognosis in OS and disease specific survival in KIRC and PRAD, compared with those without alterations.

### Results of DNA methylation analysis

**Figure 4:**
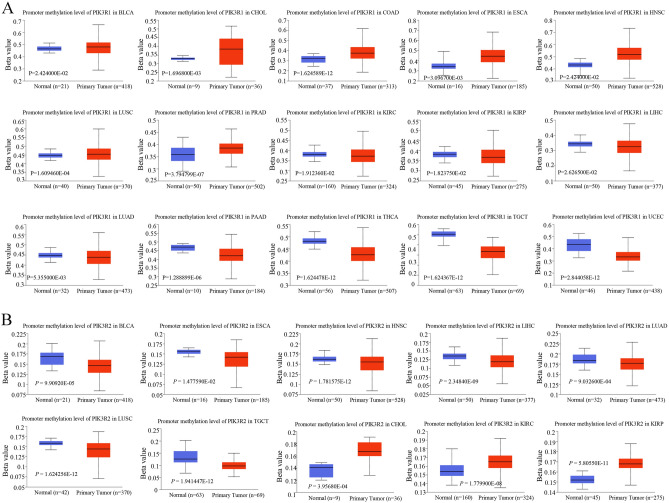
The methylation levels of PIK3R1 and PIK3R2 between normal tissues and different tumor tissues, respectively. Methylation level analysis of genes were conducted by UALCAN database (http://ualcan.path.uab.edu/analysis-prot.html). (**A**) Statistically significant differences exist in the promoter methylation levels of PIK3R1 between normal tissues and tumor tissues including BLCA, CHOL, COAD, ESCA, HNSC, LUSC, PRAD, KIRC, KIRP, LIHC, LUAD, PAAD, TGCT, THCA and UCEC. (**B**) Statistically significant differences exist in the promoter methylation levels of PIK3R2 between normal tissues and BLCA, ESCA, HNSC, LIHC, LUAD, LUSC, TGCT, CHOL, KIRC, and KIRP.

DNA methylation, as one of the epigenetic mechanisms, is involved in the occurrence and development of tumors. Hence, eight probes and nineteen probes in PIK3R1 and PIK3R2 promoters were separately used to detect the DNA methylation levels of PIK3R1 and PIK3R2. We found that promoter methylation levels of PIK3R1 were higher in BLCA, CHOL, COAD, ESCA, HNSC, LUSC, PRAD and were lower in KIRC, KIRP, LIHC, LUAD, PAAD, TGCT, THCA, and UCEC (all *P* < 0.05), compared to corresponding normal samples (Fig. 4A). Accordingly, promoter methylation levels of PIK3R2 were lower in BLCA, ESCA, HNSC, LIHC, LUAD, LUSC, TGCT, and higher in CHOL, KIRC, KIRP than normal samples (all *P* < 0.05) (Fig. 4B).

### Correlation between gene expression and TMB, MSI and ICP genes

**Figure 5:**
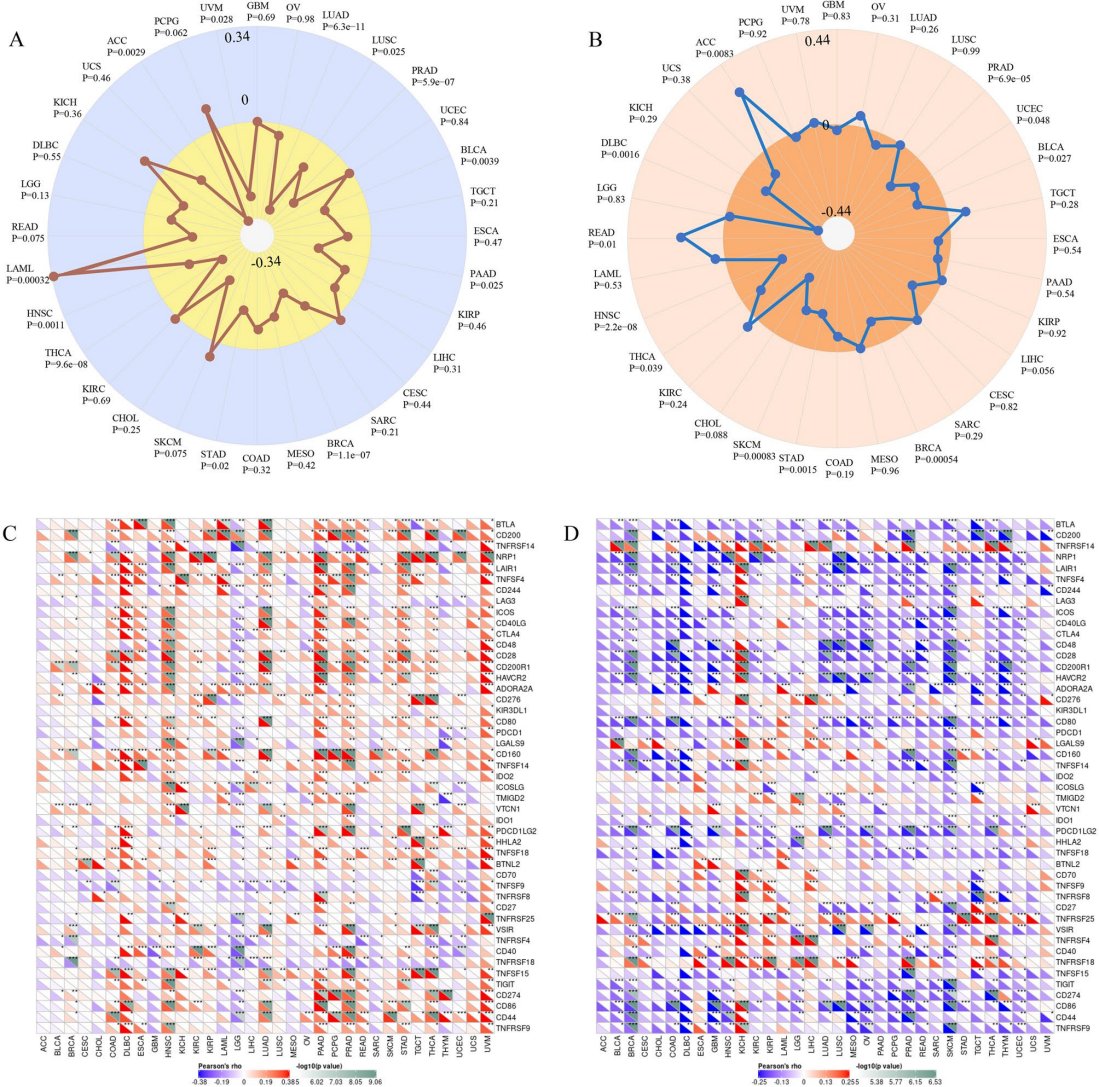
The relationship between gene expression and TMB, MSI, and ICP genes in pan-cancer. The relationship between PIK3R1 expression and TMB (**A**), MSI (**B**). Through the SangerBox tools, the Spearman rank correlation test was adopted, P < 0.05 was considered as statistically significant. The relationship between ICP genes and the expression of PIK3R1 (**C**), PIK3R2 (**D**). Analysis through the SangerBox 3.0 tools (http://sangerbox.com/Index). The lower triangle of each small square indicates the Pearson’s correlation coefficient and the upper triangle indicates the log-transformed p-value. *P < 0.05, **P < 0.01, ***P < 0.001.

Studies showed that TMB, MSI and ICP genes exert great influence on tumor immunotherapy^17,18^. Next, we explored the correlation between gene expression and TMB (Tumor mutational burden) or MSI (Microsatellite instability) in all cancers of TCGA. As shown in Fig. 5A, we found that PIK3R1 expression was negatively correlated with TMB in LUAD, LUSC, PRAD, BLCA, PAAD, BRCA, STAD, THCA, HNSC, ACC and UVM but was positively correlated with TMB in LAML (all *P* < 0.05). A significantly negative correlation was also observed between PIK3R1 and MSI in PRAD, UCEC, BLCA, BRCA, STAD, SKCM, THCA, HNSC and DLBC, while PIK3R1 expression was positively related to MSI in READ and ACC (Fig. 5B, *P* < 0.05). Subsequently, correlations with PIK3R1 expression among 47 ICP genes were found in many tumor types, especially in ESCA, PAAD, PRAD, STAD, HNSC, THCA, DLBC and UVM, and PIK3R1 expression was positively associated with more than 25 ICP genes (Fig. 5C, *P* < 0.05). Moreover, the negatively correlation between PIK3R2 expression and ICP genes existed in most tumors, but the expression of more than 20 ICP genes increased with elevated PIK3R2 expression in KICH (Fig. 5D). These results indicate that PIK3R1 and PIK3R2 might coordinate the activity of these ICP genes in different pathways and may be ideal targets for immunotherapy in specific tumor types. Unfortunately, we were only able to explore the connection between PIK3R2 expression and ICP due to lack of corresponding data.

### Correlation between gene expression and immune cells infiltration

**Figure 6:**
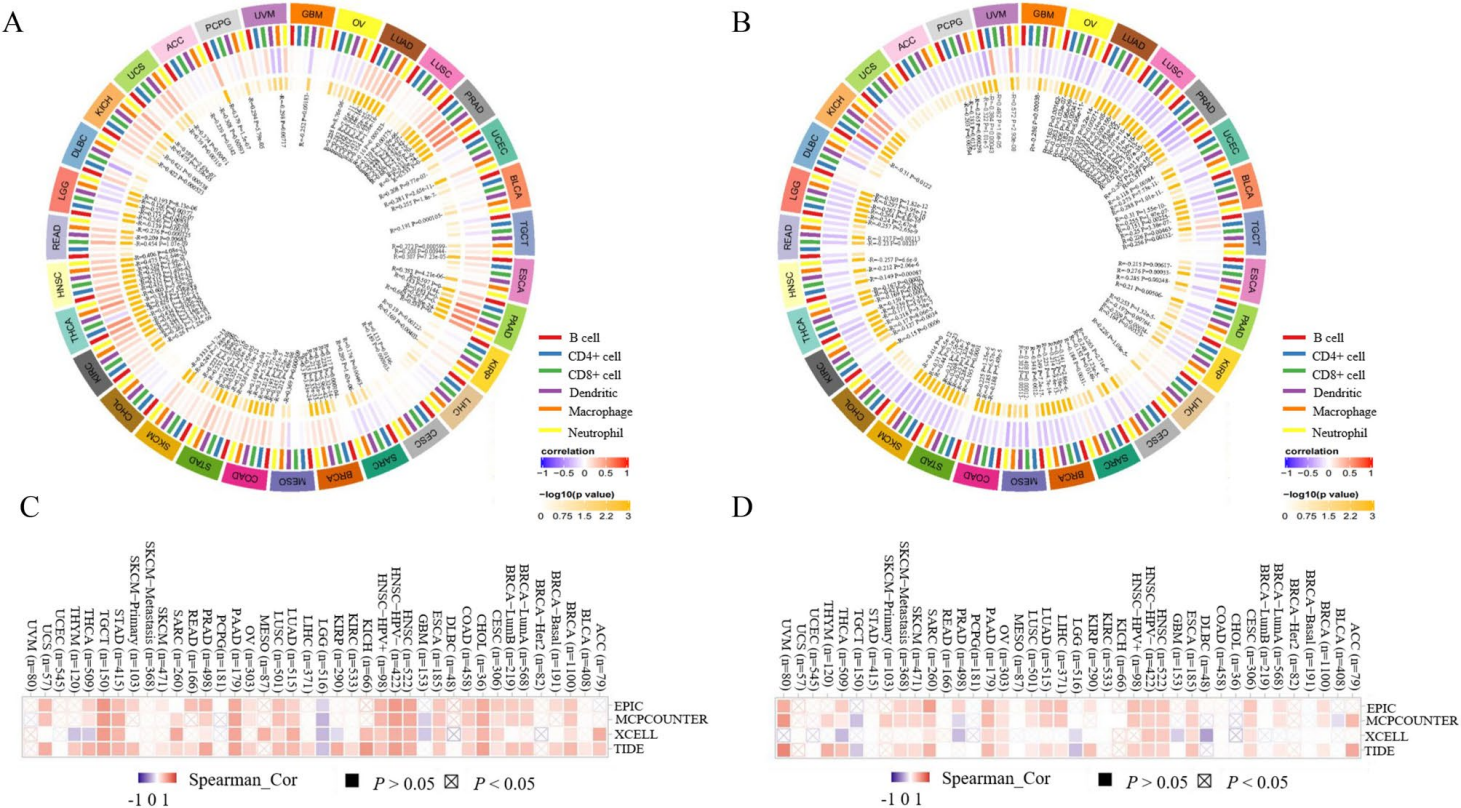
The correlation between genes expression and immune infiltration levels in pan-cancer. The correlation between immune cells including B cells, CD4 + T cells, CD8 + T cells, neutrophils, macrophages, dendritic cells and PIK3R1 expression (**A**), PIK3R2 expression (**B**). Analysis through the SangerBox 3.0 tools (http://sangerbox.com/Index). The correlation between the immune infiltration level of cancer-associated fibroblast and the expression level of PIK3R1 (**C**), PIK3R2 (**D**). Analysis through the TIMER 2.0 (http://timer.cistrome.org/database). EPIC, MCPCOUNTER, XCELL, TIDE algorithms were utilized to analyze the correlation between genes expression and immune infiltration level of cancer-associated fibroblast. P < 0.05 was considered as statistically significant.

Then, we explored whether gene expression is related to the immune infiltration level in different types of cancer. The results indicated that PIK3R1 expression was positively associated with the infiltration level of immune cells in the majority of tumors (Fig. 6A), by contrast, PIK3R2 expression was negatively associated with immune infiltration level in most cancer (Fig. 6B). In general, PIK3R1 expression represented the most significantly correlated with immune infiltration levels in BRCA, COAD and HNSC (Fig. S4A), in the meanwhile, PIK3R2 expression represented the most significantly correlated immune infiltration levels in BRCA, LGG and LUAD (Fig. S4B). Additionally, cancer-associated fibroblasts, which are critically involved in tumor progression^19^, showed significantly positive correlation with PIK3R1 expression in BRCA, CESC, CHOL, COAD, ESCA, HNSC, LUAD, LUSC, PAAD, PRAD, STAD and TGCT, while observed a negative correlation in LGG (Fig. 6C). On the contrary, PIK3R2 expression in most tumors was positively related to cancer-associated fibroblasts (Fig. 6D).

### Enrichment analysis of PIK3R1 and PIK3R2 related genes

**Figure 7:**
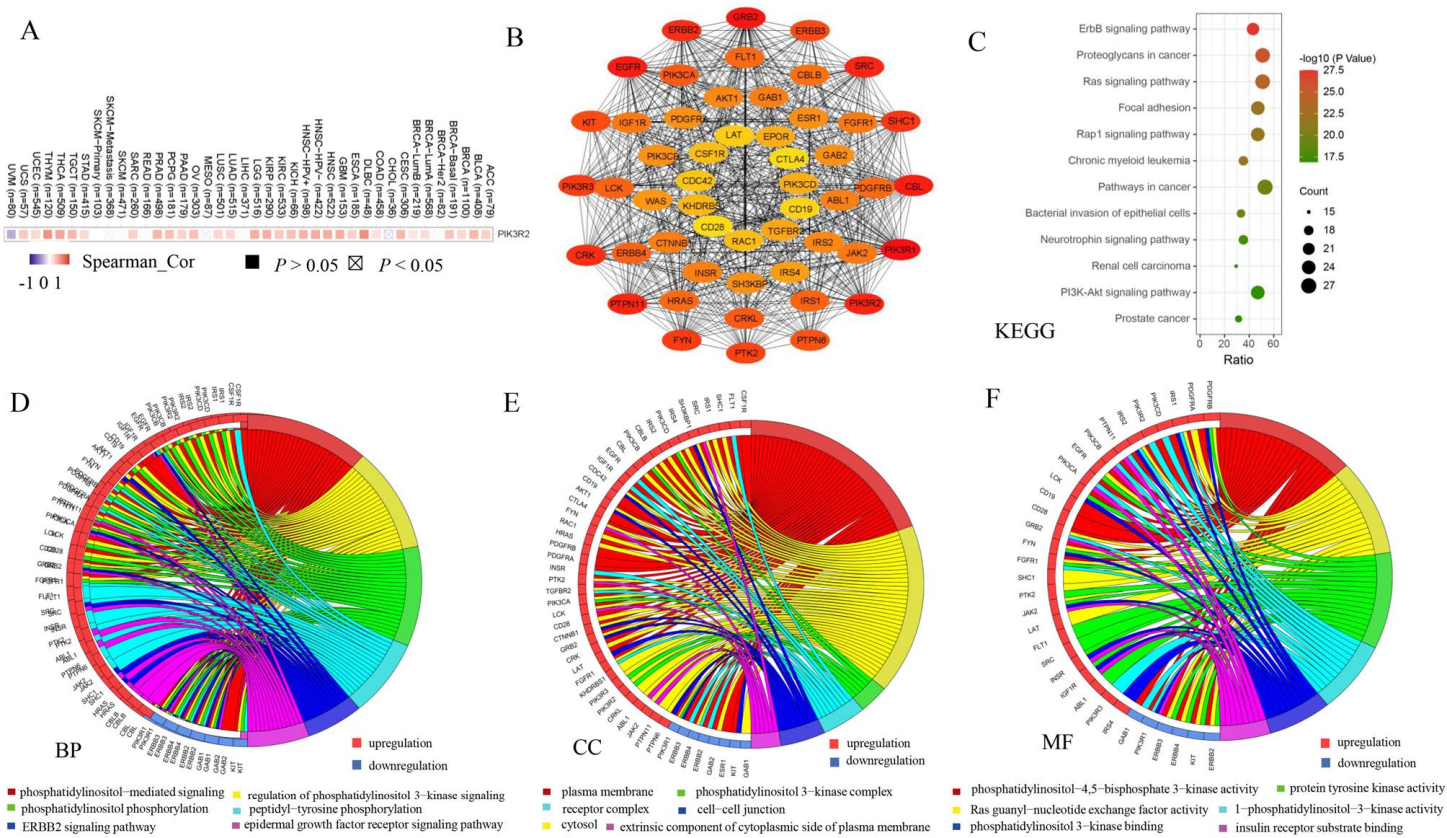
Enrichment analysis of the related genes of PIK3R1 and PIK3R2. (**A**) Heatmap indicated the relationship between PIK3R1 expression and PIK3R2 expression in tumor signaling. Analysis through the TIMER 2.0 (http://timer.cistrome.org/database). (**B**) The protein interaction network of 52 genes. These 52 genes were obtained from STRING tool (https://string-db.org/) and the protein interaction network was modified by Cytoscape 3.9.1 software (https://cytoscape.org/). Color nodes represent identified proteins, and colorful bands with different widths represent the degree of interaction between nodes. The darker the color, the stronger the interaction. (**C**) The most highly enriched items of KEGG pathway of the related genes of PIK3R1 and PIK3R2. The most highly enriched items of BP (**D**), CC (**E**), MF (**F**) of the related genes of PIK3R1 and PIK3R2 in head and neck cancer. The functional enrichment of genes interacting with PIK3R1 and PIK3R2 by the analysis of GO and KEGG by DAVID tools (https://david.ncifcrf.gov) and then the results were visualized with R 4.0.5 (https://www.r-project.org/).

We found that the PIK3R1 expression level was positively related to PIK3R2 (Fig. 7A), thus we further screened out the proteins interacting with PIK3R1 and PIK3R2 through STRING online tool to explore their molecular mechanism in tumorigenesis. We found out a total of 52 proteins that were supported by experimental evidence and the interaction network of these 52 genes was displayed in Fig. 7B. Subsequently, we utilized the gene set to perform GO (Gene ontology) and KEGG (Kyoto Encyclopedia of Genes and Genomes) pathway enrichment analyses via the DAVID online tool, and the most highly enriched items of BP, CC, MF were shown in Fig. S5 and KEGG pathway were shown in Fig. 7C. By summarizing the analysis results of this study, we found that PIK3R1 and PIK3R2 may play important roles in the occurrence and development of head and neck cancer, and there are few studies focusing on the significance of the two genes in this cancer. Therefore, we performed the GO analysis again in combination with the expression status of co-expressed genes in HNSC. The KEGG pathway results suggested that “ERBB signaling pathway”, “Proteoglycans in cancer”, “Ras signaling pathway” and “PI3K-Akt signaling pathway” may participate in the effect of PIK3R1 and PIK3R2 in tumorigenesis (Fig. 7C). GO analysis showed that the genes were highly enriched in pathways such as phosphatidylinositol-mediated signaling, plasma membrane and phosphatidylinositol-4,5-bisphosphate 3-kinase activity in BP, CC and MF, respectively (Fig. 7D–F).

## Discussion

PI3K enzymes are pivotal effectors of a series of extracellular stimuli and mediate activation of signal transduction events regulating cell survival, proliferation and migration^3^. PIK3R1 and PIK3R2 are the main regulatory subunits of PI3K. PIK3R1 encodes the regulatory subunit P85 of class I PI3K and mediates the association of RTKs with the catalytic activity of p110^20^. And PIK3R2 encodes the regulatory p85β and the expression of it induces oncogenic transformation of primary fibroblasts^21^.

It has been reported that aberrations of members along the PI3K pathway are among the most frequent driver events across different cancer lineages. As one of the core members of PI3K pathway, PIK3R1 mutation is the 12th most commonly mutated gene across cancer lineages. In the past, a lot of research focused on characterize the functional consequence of PIK3R1 mutations and found that a subset of PIK3R1 mutations were considered functional and targeting these driver mutations has the potential to benefit tumor patients^22^. A growing number of studies have shown that PIK3R1, which could regulate cancer cell proliferation, have been identified to play important roles in many human tumors carcinogenesis and implicated in tumor progression and metastasis. Unlike PIK3R1 with a high mutation rate, PIK3R2, as a ubiquitous subtype, its role has so far been ignored. But PIK3R2 expression levels were found to be elevated in advanced cancer stages of colon and breast cancers and were associated with tumor progression^23^. Moreover, it was reported that PIK3R1 serves as a tumor-suppressor in most tumors, whereas PIK3R2 acts as a tumor driver^5^. However, the role of PIK3R1 or PIK3R2 in some other cancers and whether PIK3R1 or PIK3R2 can play a role in the pathogenesis of different tumors through certain common molecular mechanisms remains to be explored. Through literature review, we failed to retrieve any publication with a pan-cancer analysis of PIK3R1 and PIK3R2 from the perspective of overall tumors. Thus, a comprehensively understanding of the function of PIK3R1 and PIK3R2 in tumors will be necessary to develop better therapies and so we performed a pan-cancer analysis of them. Our comprehensively pan-cancer analysis included a group of factors, such as gene expression levels, prognostic value, genetic alteration, immune infiltration, and relevant cellular pathway, to explore the potential molecular mechanism of PIK3R1 and PIK3R2 in pathogenesis or clinical prognosis of different tumors.

With advances in high-throughput technology and the availability of massive amounts of cancer data, bioinformatics provides a platform to explore early diagnostic or prognostic markers and potential molecular mechanisms of cancer^24^. Therefore, the availability of public databases such as TCGA, which contain functional genomics of different tumors, and bioinformatic tools allow us to perform pan-caner analysis. In the first step of our research, we utilized the TIMER and GEPIA database to determine the expression level of PIK3R1 and PIK3R2 in tumors and corresponding normal tissues. Expression of PIK3R1 was found to decrease in most tumors from TCGA, which was in accordance with the assumption that PIK3R1 has tumor suppressive feature. Moreover, decreased PIK3R1 expression was possibly associated with the dysregulation of PI3K pathway and increased signaling activation. As reported in a mice model, loss of PIK3R1 in the liver led to progressive changes of liver pathology and gradually developed into hepatocellular carcinoma with lung metastasis^25^. Additionally, our results showed that low expression of PIK3R1 was correlated with the poor prognosis of patients with various cancer types, for instance, BRCA, which was consistent with previous study^26^, and HNSC, STAD, UCEC, LUAD, etc. Noteworthily, although we failed to observe the statistical difference of PIK3R1 between LIHC and normal tissues, we found PIK3R1 underexpression was correlated to poor OS, RFS, PFS, DSS in LIHC cases through Kaplan-Meier plotter database. Moreover, another study has found that PIK3R1 was highly expressed in the majority of hepatocellular carcinoma clinical tissue specimens, and overexpression of PIK3R1 contributed to hepatocellular carcinoma progression^27^. Therefore, the expression pattern and prognostic value of PIK3R1 in hepatocellular carcinoma deserve further exploration based on larger sample sizes and clinical data. In contrast to PIK3R1, PIK3R2 was highly expressed in most tumor types, such as BRCA and COAD. And in Isabel Cortés et al.'s study^23^ of colorectal and breast cancers, PIK3R2 expression levels were also elevated in nearly half of the tumor samples and the gene was thought to regulate tumor progression. Moreover, we found high expression of PIK3R2 could generally predict poor prognosis of patients with LIHC, HNSC, KIRC, SARC and so on. These observations of pan-cancer analysis further confirmed that PIK3R1 as well as PIK3R2 may act as opposing roles in cancers.

It is well documented that the mutations of PIK3R1 and PIKR2 frequently occur in cancer lineages and they are the components of PI3K pathway, which contributes to the occurrence and development of tumor^2^. In this study, the highest frequency of PIK3R1 and PIK3R2 mutations concurrently appeared in UCEC. PIK3R1 and PIK3R2 mutations in endometrial cancer have been reported to destroy the mechanism of a pathway which regulates PTEN stability through disruption of p85α subunits, also suggesting the significance of p85 and PTEN interactions in human tumors^28^. Furthermore, we first provided evidence of the relationship between PIK3R1 expression and TMB or MSI in tumors.

We also presented comprehensive evidence of the relationship between PIK3R1 as well as PIK3R2 and immune infiltration levels of six immune cells and cancer-associated fibroblasts in TCGA tumors. There have been reports that the deletion of p85α (encoded by PIK3R1) result in the partial defect in the development, and proliferation of B cell and increased T cell proliferation was demonstrated in mice with PIK3R2 deleted^29^. Similar to these previous studies, our study showed that PIK3R1 and PIK3R2 expression were positively and negatively correlated with the level of immune infiltration in multiple tumors, respectively. Our findings also suggested PIK3R1 and PIK3R2 expression both had positive correlations with cancer-associated fibroblasts in the majority of tumors. These findings indicated that PIK3R1 and PIK3R2 may be essential for the regulation of immune infiltrating cells in assorted tumors. The relevant functional enrichment analysis of co-expressed genes with PIK3R1 and PIK3R2 were also conducted. The GO enrichment items further showed that great majority genes were involved in a series of biological processes and functions related to phosphatidylinositol 3- kinase, which is a critical regulatory node in growth-factor signaling which is of great significance in insulin signaling, cell growth, immunity and other physiological processes^20^. Furthermore, studies have suggested that abnormalities in the PI3K/AKT pathway could be the driving events in the development and progression of HNSC, and similar findings presented in our KEGG pathway analysis.

With great pleasure, we found a few noteworthy results concerning the two genes in HNSC. Comparison of PIK3R1 and PIK3R2 expression levels demonstrated obvious decrease in PIK3R1 expression level and rise in PIK3R2 level in HNSC, and both of them were significantly different from those in normal tissues. In addition, lower expression of PIK3R1and lower expression of PIK3R2 were both statistically correlated with poor OS. Besides, at multi probes in promoter region, there was a positive association of the methylation of PIK3R1 and its expression but a negative association of the methylation of PIK3R2 and its expression. In brief, our findings from multiple perspectives demonstrated several meaningful and noteworthy results in HNSC, whereas, the role of the two genes in HNSC were rarely reported. We believed that aberrations of PIK3R1 and PIK3R2, such as genetic alterations or changes in expression levels, may play a role in the progression of HNSC and can be regarded as new predictive biomarkers for the prognosis of HNSC patients.

Through our pan-cancer analysis of PIK3R1 and PIK3R2, this study revealed the important role of their aberrant expression in carcinogenesis and patient survival that warrant further investigation. However, this study still had some limitations. First, although we used different databases to analyze the possible function of genes in pan-cancer from multiple perspectives through bioinformatics methods, in vivo or invitro experiments were not performed. Future studies on the mechanism of PIK3R1 and PIK3R2 at the cellular and molecular levels may be more helpful to clarify the role of them. Second, through combined analysis of multiple databases helps us to better understand the role of PIK3R1 and PIK3R2 in tumorigenesis, and although the original data of these databases were mainly derived from TCGA database, the methods of collecting and processing data may not be consistent from database to database, which might cause systematic bias.

In summary, our comprehensive pan-cancer analysis of PIK3R1 and PIK3R2 demonstrated that PIK3R1 was frequently underexpressed while PIK3R2 was frequently overexpressed in most tumors, and they were both closely associated with clinical prognosis, DNA methylation and immune infiltration level for multiple human tumors, which greatly contribute to understanding the roles of PIK3R1 and PIK3R2 in tumorigenesis.

## Supplementary Information

Supplementary Figures.

Supplementary Tables.
