# Supplementary material for: Pan-cancer analysis on the role of PIK3R1 and PIK3R2 in human tumors
Source: Sci Rep. 2022 Apr 8;12:5924. doi: 10.1038/s41598-022-09889-0 (PMC8993854; doi:10.1038/s41598-022-09889-0)
Supplement: Supplementary file 1 — Supplementary Figures. [file 41598_2022_9889_MOESM1_ESM.docx]

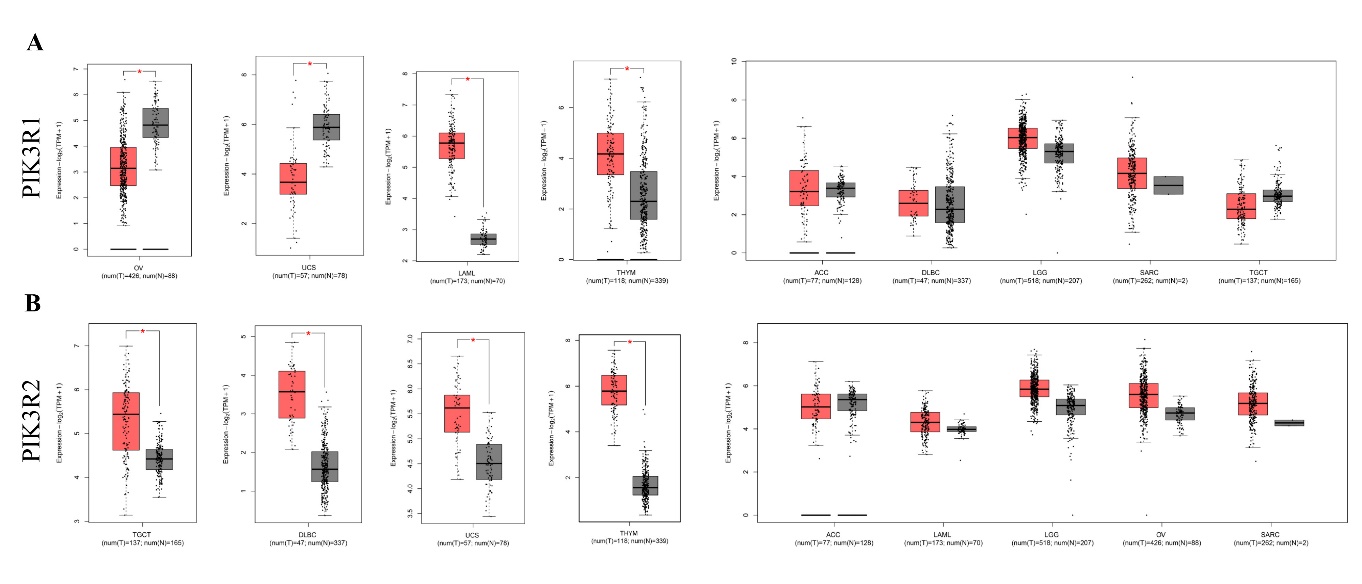


**Figure S1**| Expression level of PIK3R1 and PIK3R2 in different tumors. **(A)** Expression level of PIK3R1 in OV, UCS, LAML, THYM, ACC, DLBC, LGG, SARC, TGCT of TCGA was compared with the corresponding normal tissues in GTEX data via GEPIA database. **p* < 0.05. **(B)** Expression level of PIK3R2 in TGCT, DLBC, UCS, THYM, ACC, LAML, LGG, SARC of TCGA was compared with the corresponding normal tissues in GTE data via GEPIA database, **p* < 0.05.


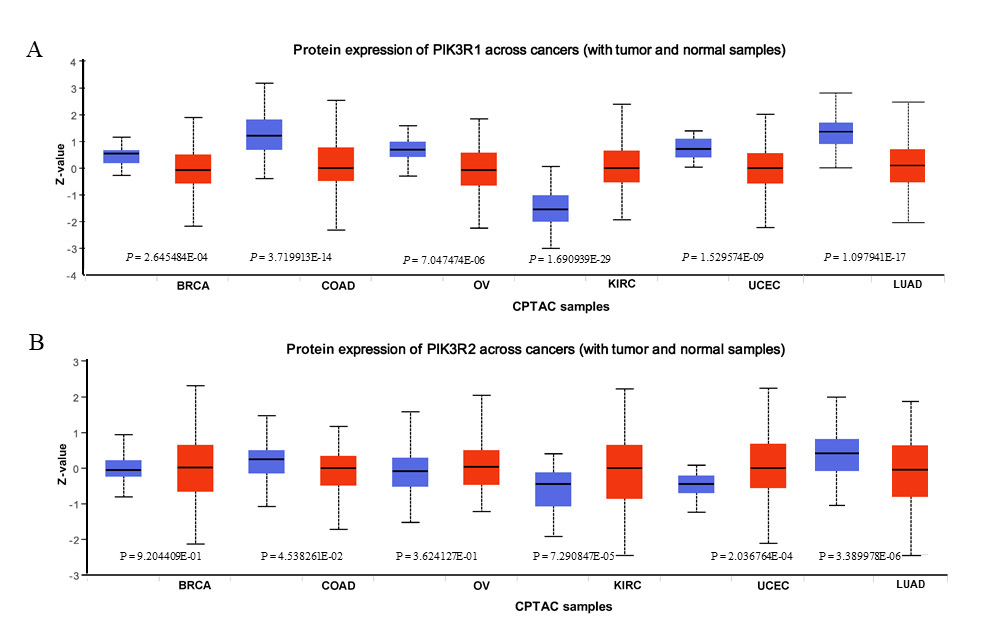


**Figure S2** | Proteomic expression levels of PIK3R1 and PIK3R2 in different tumors. **(A)** Protein expression of PIK3R1 was compared with normal samples and BRCA, COAD, OV, KIRC, UCEC, LUAD by UALCAN database. **(B)** Protein expression of PIK3R2 was compared with normal samples and BRCA, COAD, OV, KIRC, UCEC, LUAD by UALCAN database.


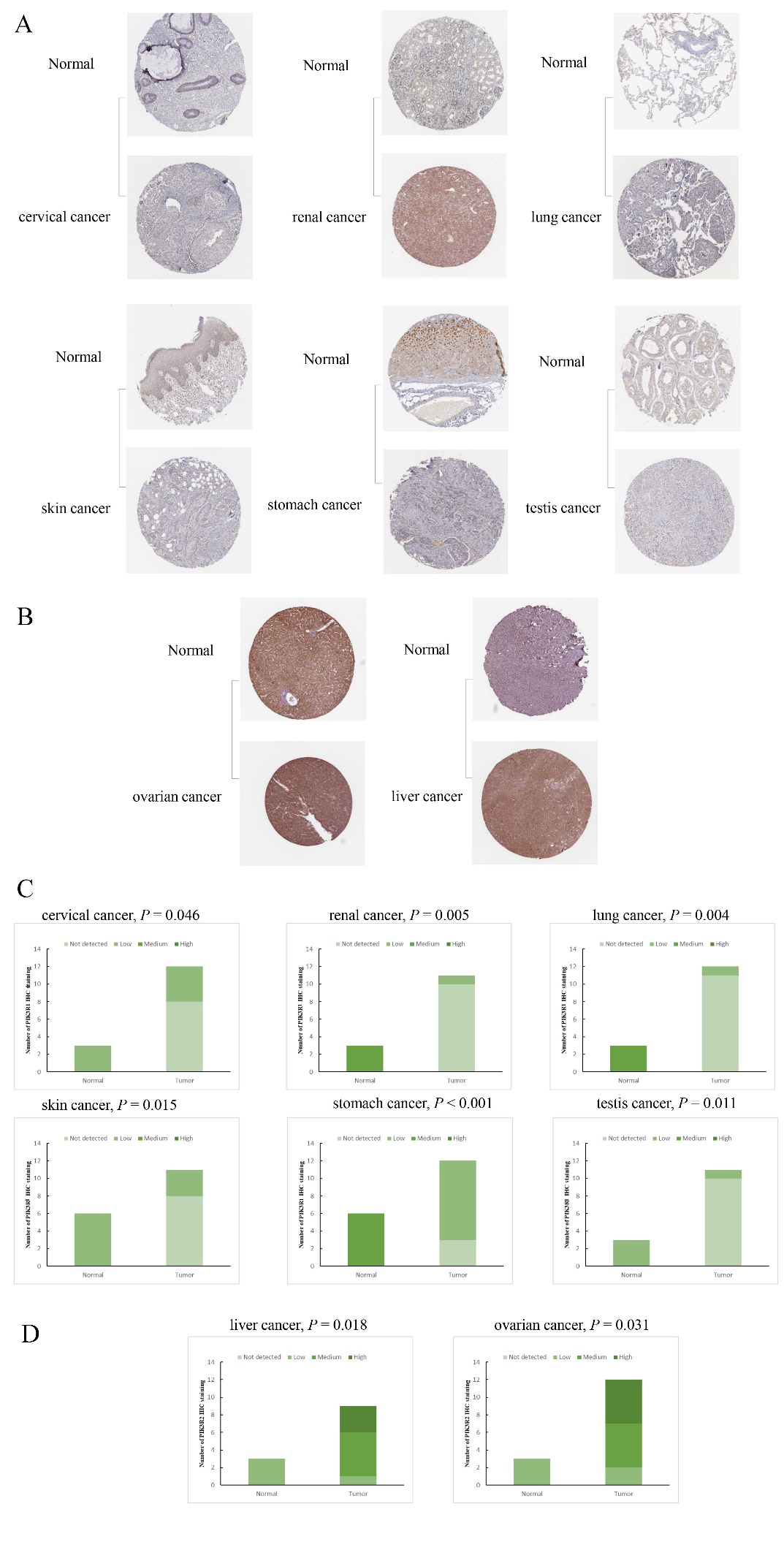


**Figure S3** | The protein expression of PIK3R1 and PIK3R2 in normal and cancer tissues. **(A)** Immunohistochemistry images of PIK3R1 in cervical cancer, renal cancer, lung cancer, skin cancer, stomach cancer and testis cancer as well as normal tissues from the HPA database. **(B)** Immunohistochemistry images of PIK3R2 in liver cancer and ovarian cancer as well as normal tissues from the HPA database. **(C)** Statistical analysis of PIK3R1 immunohistochemical staining data in normal tissues and cervical cancer, renal cancer, lung cancer, skin cancer, stomach cancer, testis cancer tissues. **(D)** Statistical analysis of PIK3R2 immunohistochemical staining data in normal tissues and liver cancer, ovarian cancer tissues.


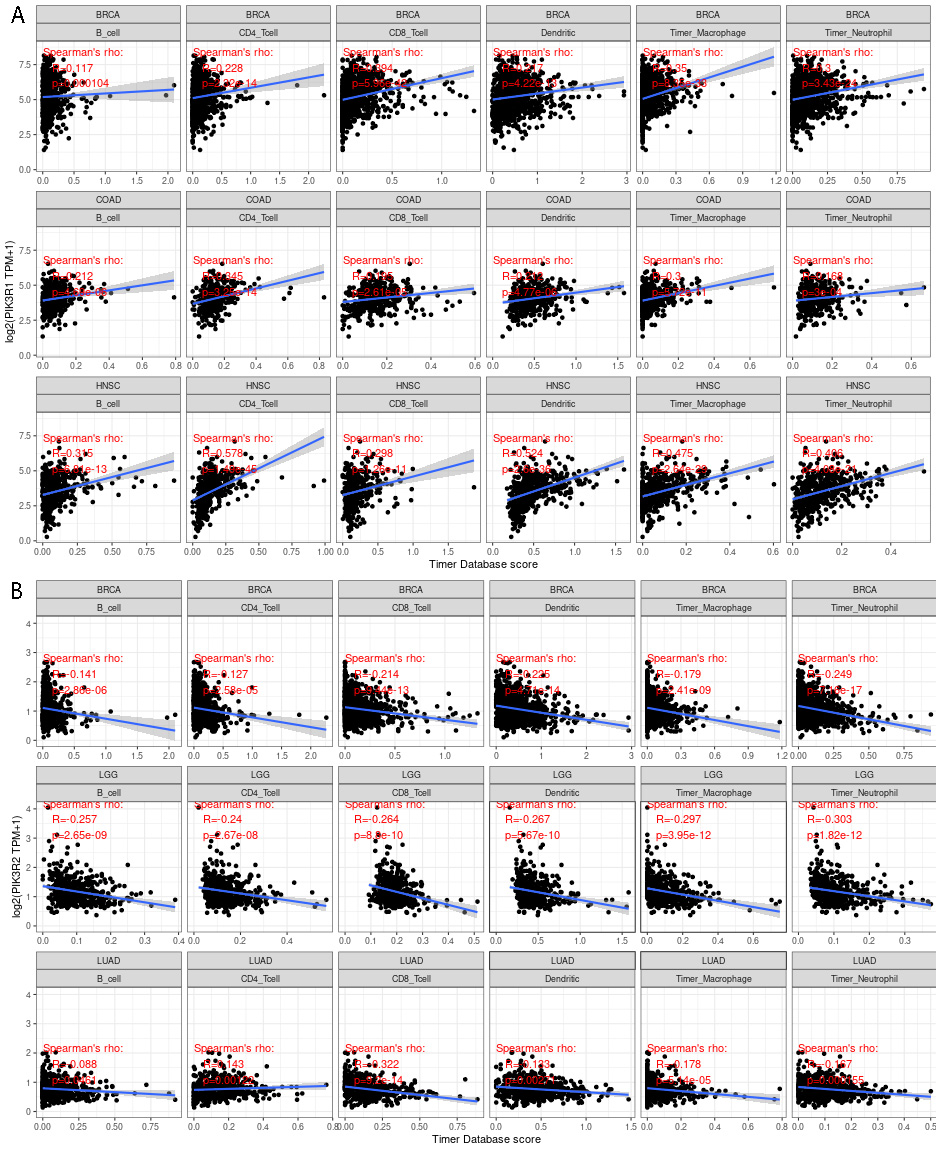


**Figure S4** | The correlation between immune infiltration level and the expression of PIK3R1 and PIK3R2 in the top three tumors. **(A)** Correlation of PIK3R1 expression with immune infiltration level in BRCA, COAD, HNSC. **(B)** Correlation of PIK3R2 expression with immune infiltration level in BRCA, LGG, LUAD.


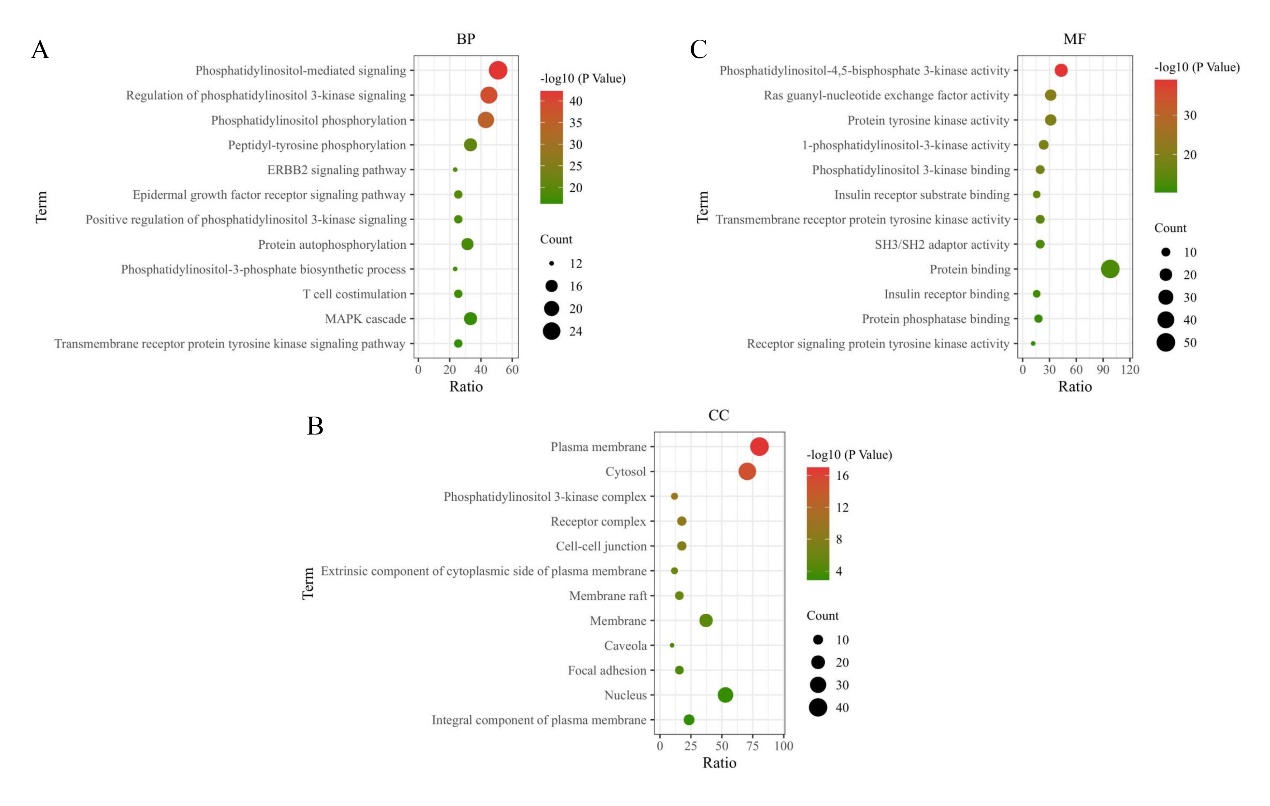


**Figure S5** | GO analysis of the related genes of PIK3R1 and PIK3R2. We performed the GO enrichment analysis on 52 co-expressed genes via DAVID tool. The bubble chart showed the most highly enriched items of BP **(A)**, CC **(B)**, MF **(C)** in tumors, respectively.
