# Supplementary material for: Pan-cancer analysis on the role of PIK3R1 and PIK3R2 in human tumors
Source: Sci Rep. 2022 Apr 8;12:5924. doi: 10.1038/s41598-022-09889-0 (PMC8993854; doi:10.1038/s41598-022-09889-0)
Supplement: Supplementary file 2 — Supplementary Tables. [file 41598_2022_9889_MOESM2_ESM.docx]

**Table S1. The detailed clinical information for tumor patients in TCGA cohort.**

| TCGA cohort | normal sample size | Tumor  sample size | Age (year， mean ± SD) | Gender (%) | Race (%) | | | Stage (%) | | | | Vital status (%) | OS time [day, M (P25, P75)] |
| --- | --- | --- | --- | --- | --- | --- | --- | --- | --- | --- | --- | --- | --- |
|  |  |  |  | Female | Asian | White | Black | Ι | Ⅱ | Ⅲ | Ⅳ | Dead |  |
| ACC | 4 | 91 | 47.03±16.35 | 59(64.8) | 2(2.2) | 77(84.6) | 1(1.1) | 9(9.9) | 44(48.4) | 19(20.9) | 17(18.7) | 33(36.2) | 1194(639,2066) |
| BLCA | 36 | 420 | 67.70±10.43 | 109(26.0) | 43(10.2) | 337(80.2) | 22(5.2) | 5(1.2) | 129(30.7) | 147(0.35) | 137(32.6) | 185(44.0) | 532(323,979) |
| BRCA | 161 | 1131 | 58.06±12.83 | 1119(98.9) | 58(5.1) | 802(70.9) | 184(16.3) | 192(17.0) | 646(57.1) | 250(22.1) | 32(2.8) | 159(14.1) | 865(471,1673) |
| CESC | 8 | 296 | 48.07±13.73 | 296(100) | 19(6.4) | 207(67.0) | 31(10.5) | - | - | - | - | 73(24.7) | 693(409,1326) |
| CHOL | 17 | 45 | 63.64±12.17 | 25(55.6) | 3(6.7) | 38(84.4) | 3(6.7) | 20(44.4) | 11(24.4) | 4(8.9) | 10(22.2) | 22(48.9) | 656(402,1077) |
| COAD | 85 | 482 | 66.75±12.70 | 225(46.7) | 11(2.3) | 245(50.8) | 67(13.9) | 83(17.2) | 174(36.1) | 90(18.7) | 137(28.4) | 98(20.3) | 700(406,1094) |
| DLBC | * | 51 | 56.76±13.68 | 27(52.9) | 18(35.3) | 32(62.7) | 1(2.0) | - | - | - | - | 12(23.5) | 946(574,1678) |
| ESCA | 64 | 185 | 62.26±11.73 | 27(14.6) | 46(24.9) | 113(61.1) | 0(0) | 18(9.7) | 78(42.2) | 56(30.3) | 9(4.9) | 77(41.6) | 400(232,681) |
| GBM | 29 | 644 | 57.60±14.34 | 253(39.3) | 13(2.0) | 551(85.6) | 54(8.4) | - | - | - | - | 537(83.4) | 372(181,625) |
| HNSC | 82 | 529 | 60.91±11.91 | 143(27.0) | 11(2.1) | 454(85.8) | 48(9.1) | 27(5.1) | 73(13.8) | 82(15.5) | 272(51.4) | 225(42.5) | 642(377,1172) |
| KICH | 70 | 112 | 50.99±13.74 | 51(45.4) | 4(3.6) | 94(83.9) | 12(10.7) | 53(47.3) | 33(29.5) | 19(17.0) | 7(6.3) | 12(10.7) | 1471(765,2941) |
| KIRC | 440 | 549 | 60.56±11.96 | 188(34.2) | 8(1.5) | 479(87.2) | 55(10.0) | 275(50.1) | 60(10.9) | 128(23.3) | 83(15.1) | 175(31.9) | 1170(563,1905) |
| KIRP | 88 | 290 | 61.43±11.99 | 77(26.6) | 6(2.1) | 208(71.7) | 62(21.4) | 174(60.0) | 21(7.2) | 50(17.2) | 15(5.2) | 44(15.2) | 770(430,1507) |
| LAML | * | 176 | 54.29±16.17 | 81(16.0) | 2(1.1) | 159(90.3) | 13(7.4) | - | - | - | - | 111(63.1) | 366(205,792) |
| LGG | * | 541 | 42.55±13.31 | 246(45.5) | 8(1.5) | 500(92.4) | 22(4.1) | - | - | - | - | 146(27.0) | 722(418,1279) |
| LIHC | 89 | 376 | 59.73±13.28 | 120(31.9) | 162(43.1) | 186(49.5) | 17(4.5) | 178(47.3) | 86(22.9) | 84(22.3) | 4(1.1) | 132(35.1) | 610(347,1146) |
| LUAD | 211 | 563 | 64.94±10.19 | 291(51.7) | 24(4.3) | 426(75.7) | 57(10.1) | 306(54.4) | 126(22.4) | 80(14.2) | 33(5.9) | 189(33.6) | 666(422,1118) |
| LUSC | 254 | 518 | 66.58±9.02 | 136(26.3) | 9(1.7) | 377(72.8) | 30(5.8) | 242(46.7) | 165(31.9) | 92(17.8) | 7(1.4) | 214(41.3) | 617(2336,1181) |
| MESO | * | 85 | 63.05±9.83 | 16(18.8) | 1(1.2) | 83(97.6) | 1(1.2) | 10(11.8) | 15(17.6) | 44(51.8) | 16(18.8) | 73(85.9) | 527(261,844) |
| OV | 125 | 618 | 59.41±11.44 | 618(100) | 24(3.9) | 529(85.6) | 34(5.5) | - | - | - | - | 370(59.9) | 1010(506,1652) |
| PAAD | 37 | 185 | 64.83±11.03 | 83(44.9) | 11(5.9) | 162(87.6) | 7(3.8) | 21(11.4) | 151(81.6) | 4(2.2) | 6(6.2) | 100(54.1) | 467(278,676) |
| PCPG | 5 | 184 | 47.26±15.15 | 102(55.4) | 6(3.3) | 153(7.1) | 20(10.9) | - | - | - | - | 8(4.3) | 752(307,1289) |
| PRAD | 118 | 513 | 60.92±6.83 | 0(0) | 12(2.3) | 422(82.3) | 64(12.5) | - | - | - | - | 10(1.9) | 910(526,1461) |
| READ | 15 | 163 | 64.05±11.65 | 72(44.2) | 1(0.6) | 82(50.3) | 6(3.7) | 33(20.2) | 47(28.8) | 48(29.4) | 25(15.3) | 29(17.8) | 638(413,1125) |
| SARC | 24 | 265 | 60.45±14.36 | 143(54.0) | 5(1.9) | 233(87.9) | 18(6.8) | - | - | - | - | 100(37.7) | 969(499,1585) |
| SKCM | * | 460 | 57.85±15.44 | 173(37.6) | 12(2.6) | 438(95.2) | 1(0.2) | 78(17.0) | 136(29.6) | 172(37.4) | 23(5.0) | 223(48.5) | 1130(504,2375) |
| STAD | 88 | 411 | 65.14±10.53 | 144(35.0) | 87(21.2) | 260(63.3) | 12(2.9) | 51(12.4) | 128(31.1) | 177(43.1) | 39(9.5) | 170(41.4) | 446(273,764) |
| TGCT | * | 139 | 31.87±9.19 | 0(0) | 4(2.9) | 124(89.6) | 6(4.3) | 106(76.3) | 12(8.6) | 14(10.1) | 0(0) | 4(2.9) | 1353(683,3243) |
| THCA | 100 | 514 | 47.22±15.80 | 374(72.8) | 52(10.1) | 338(65.8) | 27(5.3) | 290(56.4) | 52(10.1) | 113(22.0) | 57(11.1) | 16(31.1) | 941(533,1487) |
| THYM | 13 | 125 | 58.32±12.98 | 59(47.2) | 13(10.4) | 103(82.4) | 7(5.6) | - | - | - | - | 9(7.2) | 1220(717,1960) |
| UCEC | 40 | 558 | 63.95±10.96 | 558(100) | 20(3.6) | 380(68.1) | 115(2.0) | - | - | - | - | 94(16.8) | 906(523,1554) |
| UCS | 6 | 55 | 69.64±8.83 | 55(100) | 1(1.8) | 44(80.0) | 9(16.4) | - | - | - | - | 34(61.8) | 604(374,1016) |
| UVM | * | 80 | 61.65±13.95 | 35(43.8) | 0(0) | 55(68.8) | 0(0) | 0(0) | 39(48.8) | 36(45.0) | 4(5.0) | 23(28.8) | 784(446,1179) |

Note: "-" means not reported and "*" means that there was no corresponding normal control tissue for this cancer type in TCGA cohort.

**Table S2. The detailed clinical information for patients in GTEx cohort**

| **Primary site** | **Sample size** | **Gender (Female, %)** |
| --- | --- | --- |
| Adipose Tissue | 621 | 89(14.3) |
| Adrenal Gland | 161 | 69(42.9) |
| Bladder | 13 | 6(46.2) |
| Blood | 595 | 216(36.3) |
| Blood Vessel | 753 | 283(37.6) |
| Bone Marrow | 102 | 102(100) |
| Brain | 1426 | 448(31.4) |
| Breast | 221 | 94(42.5) |
| Cervix Uteri | 11 | 11(100) |
| Colon | 384 | 158(41.1) |
| Esophagus | 805 | 298(37.0) |
| Fallopian Tube | 7 | 7(100) |
| Heart | 493 | 168(34.1) |
| Kidney | 38 | 9(23.7) |
| Liver | 141 | 47(0.33) |
| Lung | 381 | 130(34.1) |
| Muscle | 478 | 174(36.4) |
| Nerve | 335 | 117(34.9) |
| Ovary | 112 | 112(100) |
| Pancreas | 203 | 82(40.4) |
| Pituitary | 126 | 37(29.4) |
| Prostate | 122 | 0(0) |
| Salivary Gland | 71 | 25(35.2) |
| Skin | 977 | 345(35.3) |
| Small Intestine | 106 | 42(39.2) |
| Spleen | 121 | 51(42.1) |
| Stomach | 209 | 89(42.6) |
| Testis | 208 | 0(0) |
| Thyroid | 366 | 135(36.9) |
| Uterus | 93 | 93(100) |
| Vagina | 99 | 99(100) |

**Table S3. The detailed clinical information for patients in CPTAC dataset**

| CPTAC | Normal  Sample size | Tumor  Sample size | Age (year,  mean±SD) | | Gender | Race (%) | | | Stage (%) | | | |
| --- | --- | --- | --- | --- | --- | --- | --- | --- | --- | --- | --- | --- |
|  |  |  | 21-60 | 61-80 | Female (%) | Caucasian | African-american | Asian | Ι | Ⅱ | Ⅲ | Ⅳ |
| BRCA | 18 | 125 | 60(48.0) | 60(48.0) | - | 80(64.0) | 18(14.4) | 20(16.0) | 4(3.2) | 74(59.2) | 32(25.6) | - |
| COAD | 100 | 97 | 34(35.1) | 60(61.9) | 56(57.7) | 71(73.2) | 7(7.2) | 16(16.5) | 10(10.3) | 39(40.2) | 40(41.2) | 8(8.2) |
| OV | 25 | 100 | 54(54.0) | 43(43.0) | 100(100.0) | 86(86.0) | 2(2.0) | 8(8.0) | 2(2.0) | 75(75.0) | 16()16.0 | - |
| KIRC | 84 | 110 | 55(50.0) | 55(50.0) | 30(27.3) | 61(55.5) | 1(0.9) | 1(0.9) | 52(47.3) | 13(11.8) | 33(30.0) | 12(10.9) |
| LUAD | 111 | 111 | 48(43.2) | 63(56.8) | 38(34.2) | 34(30.69) | 1(0.9) | 1(0.9) | 59(53.2) | 30(27.0) | 21(18.9) | 1(0.9) |
| UCEC | 31 | 100 | 37(37.0) | 63(63.0) | 100(100.0) | 58(58.0) | 3(3.0) | 1(1.0) | 74(74.0) | 8(8.0) | 15(15.0) | 3(3.0) |

Note: "-" means not reported.
